# Case Report: Primary lymphoepithelioma-like intrahepatic cholangiocarcinoma

**DOI:** 10.3389/fonc.2023.1146933

**Published:** 2023-05-01

**Authors:** Fei Liu, Qing Xu, Parbatraj Regmi, Fu-Yu Li, Yi-Xin Lin

**Affiliations:** ^1^Department of Biliary Surgery, West China Hospital of Sichuan University, Chengdu, Sichuan, China; ^2^Institute of Clinical Pathology, Key Laboratory of Transplant Engineering and Immunology, West China Hospital of Sichuan University, Chengdu, China

**Keywords:** LEL-ICC, liver, lesion, EBV – epstein-barr virus, surgical resection

## Abstract

**Background:**

Lymphoepithelioma-like intrahepatic cholangiocarcinoma (LEL-ICC) is a rare variant of intrahepatic cholangiocarcinoma. Epstein–Barr virus (EBV) infection was considered to play a pivotal role in the tumorigenesis of LEL-ICC. It is difficult to diagnosis of LEL-ICC due to the lack of specific features regarding the laboratory test results and imaging findings. At present, the diagnosis of LEL-ICC mainly depends on the histopathologic and immunohistochemical examinations. In addition, the prognosis of LEL-ICC was better than classical cholangiocarcinomas. To our knowledge, only few cases of LEL-ICC have been reported in the literature.

**Case presentation:**

We presented a case of a 32-year-old Chinese female with LEL-ICC. She had a 6-month history of upper abdominal pain. The magnetic resonance imaging (MRI) showed a 1.1× 1.3 cm lesion in the left lobe of liver, appearing low signal intensity on T1-weighted images and high signal intensity on T2-weighted images. The patient underwent laparoscopic left lateral sectionectomy. The postoperative histopathologic and immunohistochemical examinations results allowed for the definitive diagnosis of LEL-ICC. The patient was free from tumor recurrence after a 28 months follow-up.

**Conclusion:**

In this study, we reported a rare case of LEL-ICC associated with both HBV and EBV infection. EBV infection might play a pivotal role in the carcinogenesis of LEL-ICC, and surgical resection is still the most effective treatment at present. Further research on the etiology and treatment strategies of LEL-ICC is required.

## Introduction

Epstein–Barr virus (EBV) belongs to the herpesvirus family with oncogenic properties (1). EBV infection is associated with certain malignancies and was also considered to play a pivotal role in the tumorigenesis of Lymphoepithelioma-like carcinoma (LELC) (2, 3). LELC is a rare malignancy consisting of undifferentiated epithelial cells with intense lymphocytic infiltration (4). The unique tumor has been reported in various organs such as the salivary glands, gastrointestinal tract, lungs, thymus, and urinary tract (5). However, LELC is rarely identified in the liver. Hepatic LELC can be divided into two types: lymphoepithelioma-like intrahepatic cholangiocarcinoma (LEL-ICC) and lymphoepithelioma-like hepatocellular carcinoma (LEL-HCC). Preoperative diagnosis of LELC is difficult due to the lack of specific features regarding the laboratory test results and imaging findings. At present, the diagnosis of LELC mainly depends on the histopathologic and immunohistochemical examinations. To our knowledge, only few cases of LEL-ICC have been reported, and the understanding of LEL-ICC is very limited. Therefore, more reports are needed to describe the comprehensive characteristics of LEL-ICC. In this study, we presented a case of LEL-ICC with both HBV and EBV infection, which was treated by laparoscopic left lateral sectionectomy. We obtained written informed consent from the patient and the patient’s parents for the procedure and publication.

## Case report

**Figure 1:**
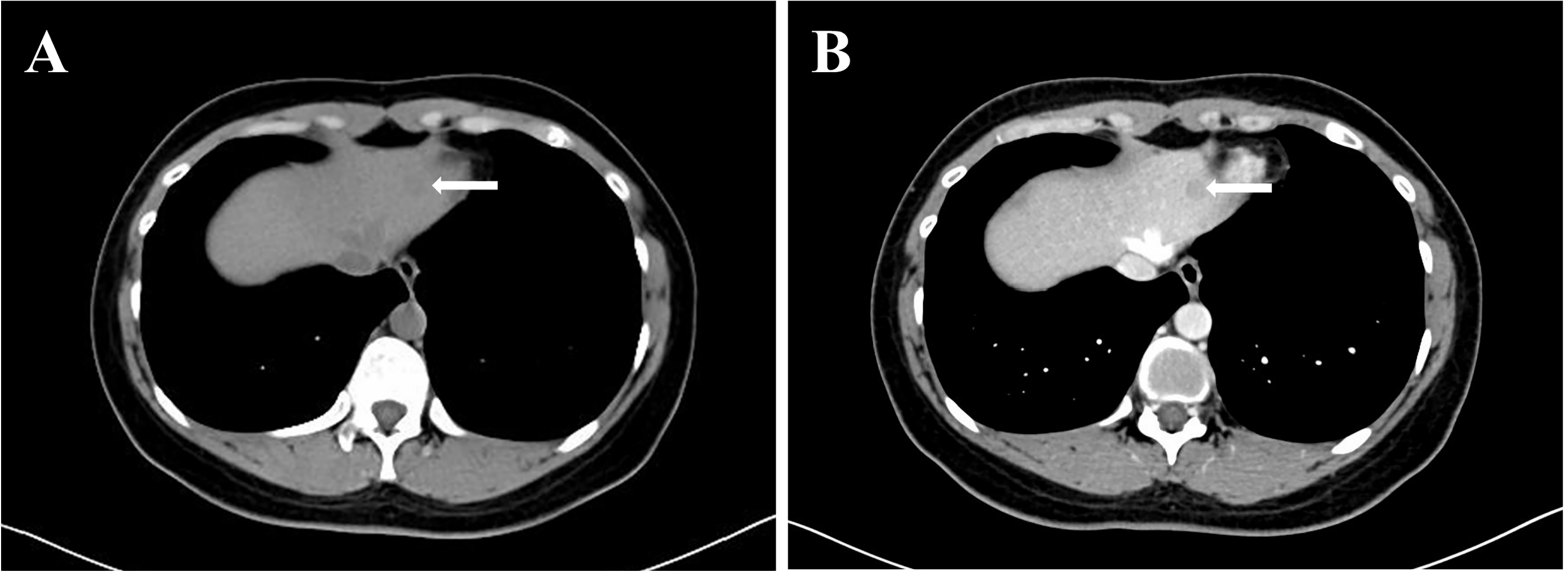
The lesion in the left lobe of liver was detected on CT.

**Figure 2:**
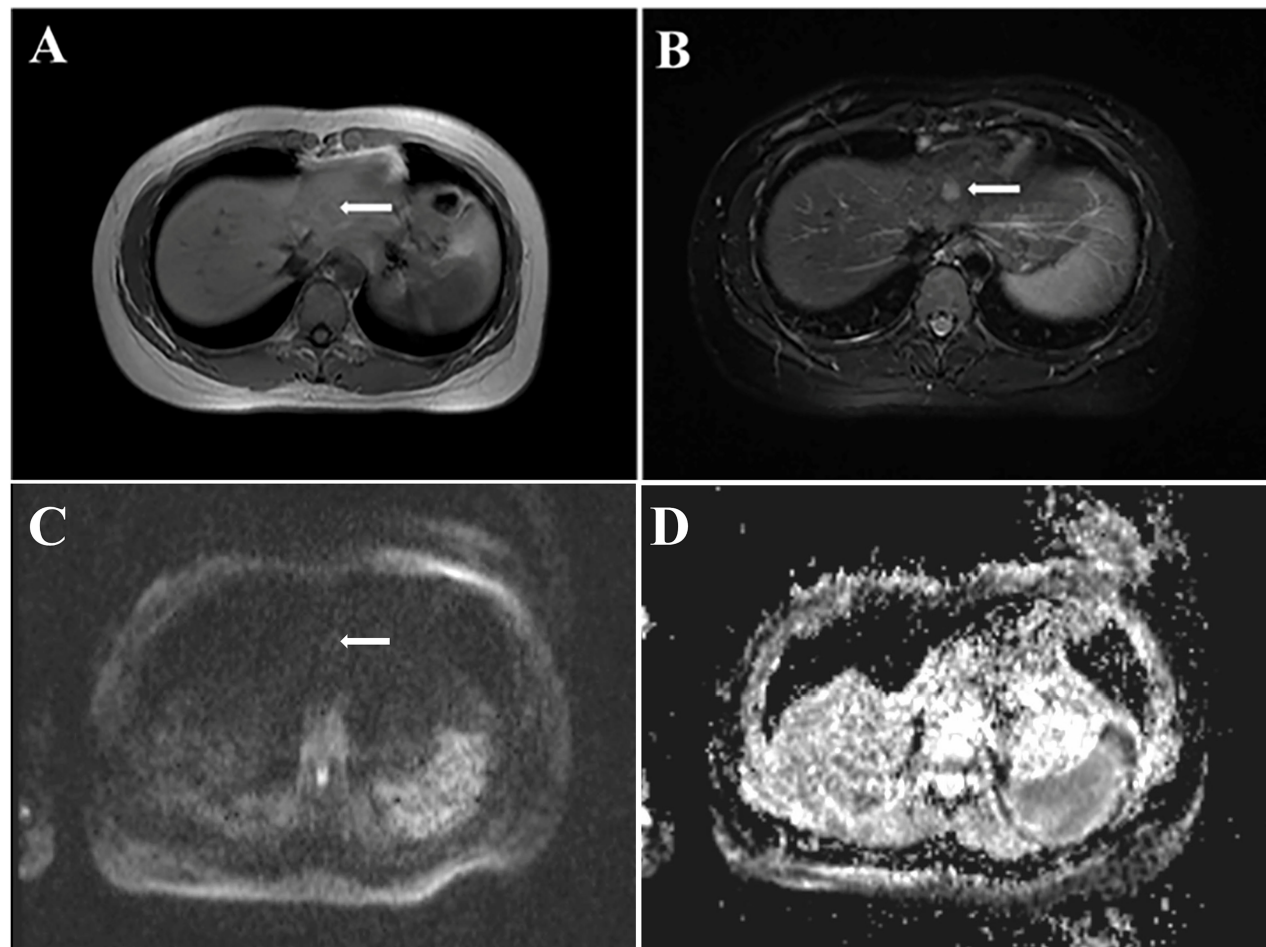
Magnetic Resonance Imaging showed a 1.1×1.3 cm lesion in the left lobe of liver. **(A)** low signal intensity on T1-weighted images; **(B)** high signal intensity on T2-weighted images; **(C)** ring enhancing on DWI image; **(D)** ADC image of the lesion (ADC value: 800).

**Figure 3:**
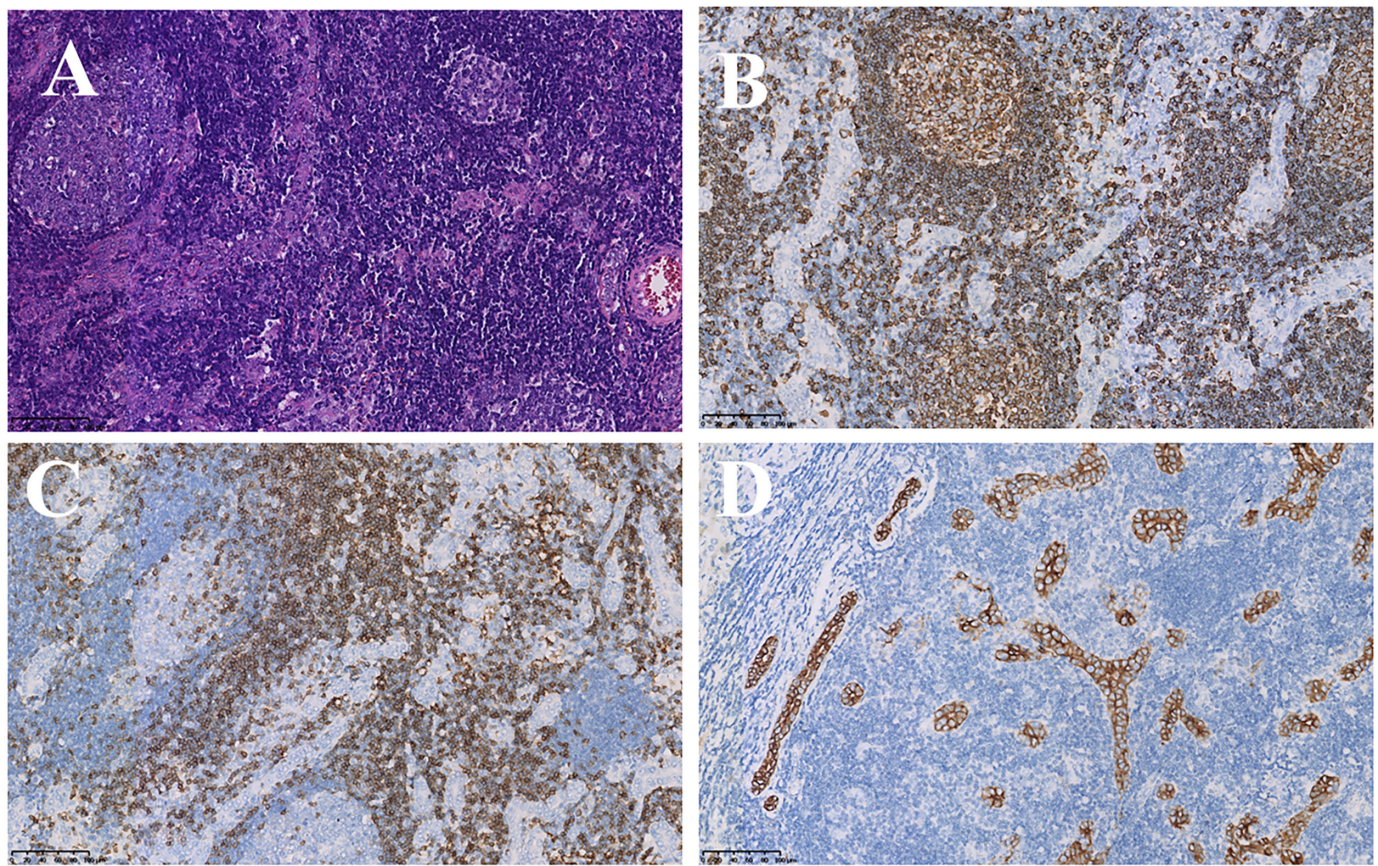
Pathological examination and immunohistochemical staining. **(A)** Tumor was composed of undifferentiated epithelial cells with some atypical glands, and significant lymphocytic infiltration (HE stain, ×20); **(B, C)** Lymphatic tissue positive for CD20 (B-cells, B, ×20), CD3 (T-cells, C, ×20); **(D)** tumor cells positive for CK7 (immunostaining, ×20).

A 32-year-old Chinese female was admitted to Sichuan University West China Hospital with a 6-month history of upper abdominal pain. She denied previous radiotherapy or industrial chemical exposure. She had one previous pregnancy and and gave birth to a boy. In addition, she denied previous hormonal treatments and contraceptives. She was found to have viral hepatitis B for 6 years and had not received any treatment. Besides, she was healthy with no relevant medical or family history of diseases, such as hypertension or diabetes, and no history of smoking or alcohol consumption. Physical examination was unremarkable. A blood count showed Hb 14.2 g/dl (13–17.5), white blood cells 7.12×10^9^/L (3.5-9.5), platelets 249×10^9^/L (100–300), total bilirubin 12.5 umol/L (5.0-28), and AST 35 IU/L (<50). Serological testing for tumor marker of carcinoembryonic antigen (CEA) was 5.54 ng/ml (CEA ≥ 3.4 ng/ml was defined as abnormal) and hepatitis B surface antigen (HBsAg) was positive. The hepatitis B virus DNA (HBV-DNA) was less than 1×10^2^ IU/ml (HBV-DNA ≥ 1×10^2^ IU/ml was defined as HBV infection active), suggesting that HBV infection was inactive. The cancer antigen19-9 (CA19-9 ≥ 30 U/ml was defined as abnormal), CA125 (CA125 ≥ 24 U/ml was defined as abnormal) and α-fetoprotein (AFP≥ 7 ng/ml was defined as abnormal) was 25.6 U/ml, 13.3U/ml and 3.37, respectively. Abdominal computed tomography (CT) showed the lesion in the left lobe of liver was detected, and no tumor was detected in any other organs (**Figure 1**). Magnetic resonance imaging (MRI) of the upper abdomen was performed in our hospital for further diagnosis. The MRI showed a 1.1×1.3 cm lesion in the left lobe of liver, appearing low signal intensity on T1-weighted images and high signal intensity on T2-weighted images (**Figure 2**). Due to the similar appearance, hepatocellular carcinoma (HCC) was considered for preoperative diagnosis. The patient eventually underwent a laparoscopic liver resection of the left lobe. Macroscopically, the tumor was a yellowish solid mass with a diameter of 12mm. Microscopically, the lesion composed of undifferentiated epithelial cells with some atypical glands, and significant lymphocytic infiltration (**Figure 3A**). The epithelial tumor cells were featured by eosinophilic cytoplasm with large nuclei and prominent nucleoli. EBVencoded RNA (EBER) *in situ* hybridization was positive in tumor tissues. In addition, immunohistochemical analysis showed the lymphatic tissue positive for CD20 (B-cells, **Figure 3B**), CD3 (T-cells, **Figure 3C**), Ki-67 and negative for IgG4. Meanwhile, tumor cells positive for CK7 (**Figure 3D**), and negative for CK20, supporting the diagnosis of LEL-ICC.

Post-operative recovery of the patient was well. The patient was discharged on postoperative day 5 with good general condition. The laboratory parameters were normal and we recommended regular follow-up in the outpatient clinic.

**Figure 4:**
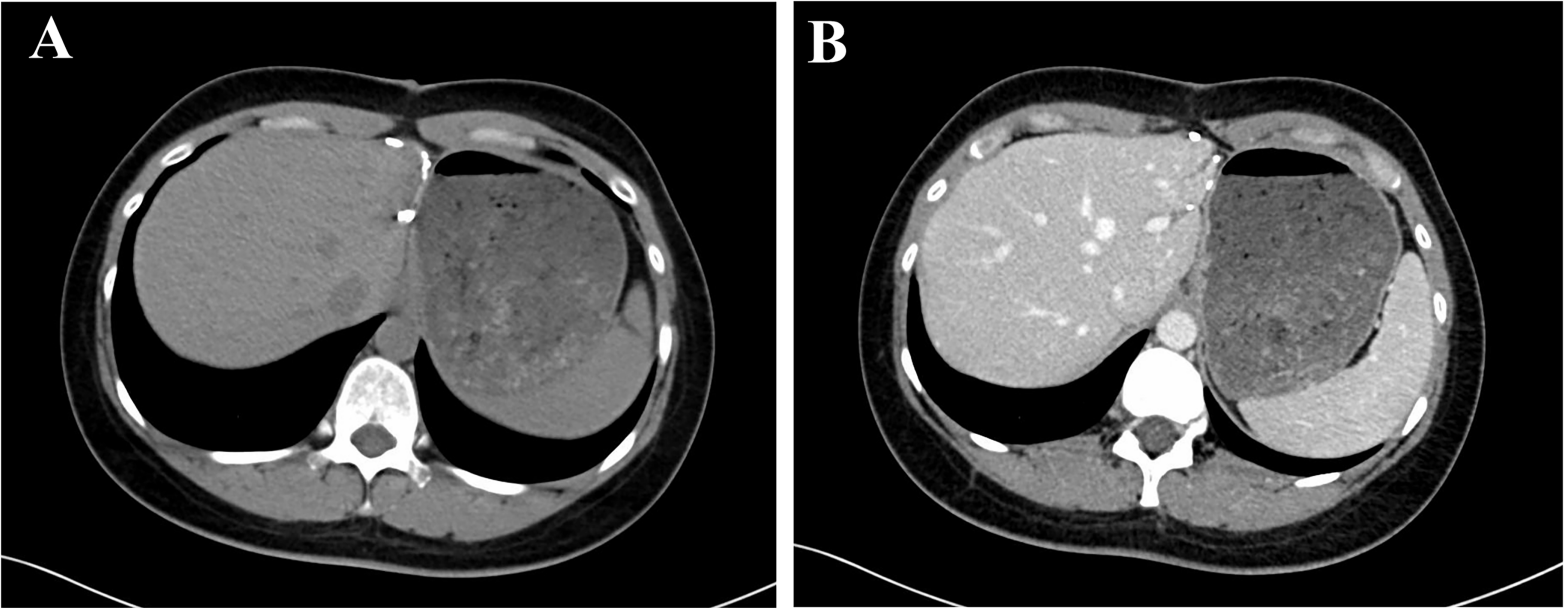
CT showed the patient was free from tumor recurrence after a 28 months follow-up.

Patients monitored the disease progression at the outpatient of our hospital every 3 months in the first two years after surgery and every 6 months thereafter *via* blood examination, ultrasonography (US), CT, and MRI. The systematic update of patients’survival information was performed once a year. The last outpatient follow-up was in August 2022, and the tumor markers were normal. The patient was free from tumor recurrence after a 28 months follow-up (**Figure 4**).

## Discussion

LELC is a rare malignancy consisting of undifferentiated epithelial cells with abundant lymphocytic infiltration (4). Up to now, it has been reported in various organs such as the salivary glands (6), gastrointestinal tract (7), lungs (8), urinary tract (9), ovaries (10), and other locations. The cause of LELC remains unclear at present. The LELC is often associated with EBV infection (approximately 70% with EBER positive *in situ* hybridization) (11). Previous studies reported that EBV was considered to play a pivotal role in the tumorigenesis of LELC (2, 3).

A recent review by Ding et al. (2) identified 92 cases of hepatic LELC, including 26 cases of LEL-ICC, and 67 cases of LEL-HCC. The mean age was 55 years old (range,19-79 years old) of the patients with 88.5% being Asian and 69.2% being female. Furthermore, 92.3% LEL-ICC patients were usually a single lesion and 73.1% patients were EBV positive. EBV infection might play a pivotal role in the carcinogenesis of LEL-ICC. However, Adachi et al. (12) reported there were no obvious histopathologic differences between EBV-negative and EBV-positive LEL-ICC. Hence, the association between EBV and LEL-ICC is still inconclusive and controversial. Further studies focusing on the etiology of LEL-ICC are also required.

Clinical presentations of LEL-ICC are nonspecific. Most patients were almost asymptomatic, and the hepatic lesion was identified during a routine health examination. Some patients have the upper abdominal pain, abdominal fullness or fever (2). The definitive diagnosis of LEL-ICC mainly depends on the histopathologic and immunohistochemical examinations, which consisted of undifferentiated epithelial cells with intense lymphocytic infiltration. The tumor cells were featured by eosinophilic cytoplasm with large nuclei and prominent nucleoli. Immunohistochemically, tumor cells are usually positive for various biliary-type cytokines, such as CK7. In our case, the epithelial tumor cells were positive for CK7, supporting the diagnosis of LEL-ICC.

The prognosis of LEL-ICC was better than classical cholangiocarcinomas (1). Notablely, Chan et al. (1) statistically analyzed seven LEL-ICC patients and found that the 5-year overall survival was 100%. The clinical outcomes were indeed generally well, with some patients alive without recurrence for 165 months (1). In our case, 28 months have passed since the surgery; the patient is still alive and has no tumor recurrence. Further analyses recruiting more patients are required to determine the prognosis of LEL-ICC. In addition, there is no consensus on the standardized treatment strategies for LEL-ICC. Almost all cases reported showed that surgical resection is still the most effective treatment at present (2). However, postoperative chemotherapy, postoperative radiotherapy, or targeted therapy was rarely adopted. It had been reported that the survival time of patient with lymph node metastasis after surgery and postoperative radiation was 54 months without recurrence (13). Hence, further analyses recruiting more patients are required to develop appropriate treatment strategies.

## Conclusions

In this study, we reported a rare case of LEL-ICC associated with both HBV and EBV infection. The patient underwent laparoscopic left lateral sectionectomy based on the preoperative diagnosis of HCC, and LEL-ICC was confirmed by histopathologic and immunohistochemical examinations. EBV infection might play a pivotal role in the carcinogenesis of LEL-ICC, and surgical resection is still the most effective treatment at present. In addition, the favorable prognosis could be generally achieved after radical resection. Further research on the etiology and treatment strategies of LEL-ICC is required.

## Data availability statement

The original contributions presented in the study are included in the article/**Supplementary Material**, Further inquiries can be directed to the corresponding authors.

## Ethics statement

Written informed consent was obtained from the individual (s) for the publication of any potentially identifiable images or data included in this article.

## Author contributions

FL and QX contributed to the data acquisition and drafted the manuscript. PR contributed to data acquisition. Y-XL and F-YL contributed to the study design and revised the manuscript. All authors contributed to the article and approved the submitted version.

## Supplementary material

Supplementary Figure 1 Dynamic images of this lesions on MRI.
